# Neurodevelopmental risk in a referred sample of hospitalized children with congenital heart disease: interrelation between communication and feeding vulnerabilities

**DOI:** 10.1016/j.clinsp.2026.101199

**Published:** 2026-10-14

**Authors:** Camila Pimentel-Piemonte, Fernanda Chiarion Sassi, Ana Paula Ritto, Gisele Chagas de Medeiros, Claudia Regina Furquim de Andrade

**Affiliations:** a Department of Physical Therapy, Speech-Language Pathology and Occupational Therapy, Faculdade de Medicina da Universidade de São Paulo (FMUSP), São Paulo, SP, Brazil; b Instituto do Coração do Hospital das Clínicas da Faculdade de Medicina da Universidade de São Paulo (InCor-HCFMUSP), São Paulo, SP, Brazil

**Keywords:** Heart defects, Congenital, Child development, Communication disorders, Deglutition disorders, Hospitalization, Speech-language pathology

## Abstract

**Objective:**

To determine the prevalence of communication developmental risk in hospitalized children with Congenital Heart Disease (CHD) and to identify clinical, surgical, and hospitalization-related factors independently associated with increased risk.

**Methods:**

This cross-sectional observational study was conducted at a tertiary pediatric cardiac center between September 2024 and April 2025. Sixty-five children referred for speech-language pathology assessment underwent communication development screening using a structured developmental observation protocol. Functional feeding status was assessed using the Pediatric Functional Oral Intake Scale. Multivariable logistic regression was used to identify factors independently associated with communication developmental risk, with Firth's penalized logistic regression performed as a sensitivity analysis given the low events-per-variable ratio.

**Results:**

Twenty-five children (38%) were classified as being at risk for delayed communication development. Hospitalization in a ward setting, compared with intensive care, was independently associated with lower odds of risk classification (adjusted OR = 0.13, 95% CI 0.02‒0.77, p = 0.025), an association confirmed by the Firth sensitivity analysis (adjusted OR = 0.18, 95% CI 0.03‒0.80, p = 0.024). Length of hospital stay, cyanotic physiology, cardiopulmonary bypass duration, and aortic cross-clamp time were not independently associated with risk. Children classified as at risk also showed significantly greater feeding restrictions.

**Conclusion:**

Communication developmental risk was common among hospitalized children with CHD. Hospitalization setting and feeding performance, rather than traditional markers of clinical severity, were associated with communication risk, suggesting a multifactorial model that integrates biological, functional, and environmental factors. These findings suggest that feeding function, alongside hospitalization setting, may help flag children at higher developmental risk before formal communication assessment is available. These exploratory findings support incorporating structured developmental surveillance into routine pediatric cardiac care.

## Highlights

• Over one-third of hospitalized children with CHD showed communication risk.
• Ward admission was linked to lower odds of communication developmental risk.
• Traditional perioperative risk factors were not independently associated.
• Feeding restrictions (pFOIS) were associated with communication risk.
• Firth regression confirmed findings despite low events-per-variable ratio.

## Introduction

Congenital Heart Disease (CHD) is the most common congenital malformation in childhood and remains a major cause of pediatric morbidity, prolonged hospitalization, and complex surgical intervention. Over recent decades, advances in fetal diagnosis, surgical techniques, cardiopulmonary bypass management, and neonatal intensive care have substantially improved survival, allowing an increasing number of children with CHD to survive into adolescence and adulthood. However, alongside improvements in cardiovascular outcomes, neurodevelopment has emerged as one of the most relevant long-term concerns among survivors of complex CHD. Current evidence indicates that these children are particularly vulnerable to cognitive, motor, behavioral, feeding, and communication impairments, which may be identified early in infancy and persist throughout development.^1^^,^^2^

Neurodevelopmental vulnerability in children with CHD is understood as the result of a dynamic interaction among biological, hemodynamic, surgical, and environmental factors. Structural and maturational brain abnormalities may originate during fetal life as a consequence of altered cerebral perfusion, including reduced brain growth, white matter abnormalities, and disrupted neural connectivity. During the postnatal period, exposure to chronic hypoxia, cyanosis, systemic inflammatory responses associated with cardiopulmonary bypass, hemodynamic instability, prolonged sedation, and mechanical ventilation may further increase developmental risk.^1^^,^^2^ In addition, children hospitalized in intensive care settings are frequently exposed to restricted mobility, repeated invasive procedures, sleep disruption, pain, and reduced opportunities for age-appropriate sensorimotor and social experiences.^3^^,^^4^ Therefore, the hospital environment has increasingly been recognized not only as a setting for clinical stabilization, but also as an environmental context capable of influencing developmental trajectories.^3^^,^^4^ Importantly, many of these biological and perioperative risk factors are closely associated with indicators of clinical severity, such as cyanotic physiology, greater anatomical complexity and surgical burden, prolonged hospitalization, and the need for intensive postoperative care. These factors frequently coexist and may have cumulative effects on neurodevelopment, as children with more complex cardiac conditions often experience longer hospital stays, greater exposure to invasive procedures, and reduced opportunities for developmentally appropriate interactions. Consequently, developmental risk in children with CHD is often shaped by the combined influence of underlying cardiac physiology, surgical burden, and the cumulative effects of hospitalization itself.^1^, ^2^, ^3^, ^4^

Among the neurodevelopmental impairments observed in children with CHD, language and communication difficulties are particularly relevant because of their early onset and potential long-term impact on learning, social participation, behavior, emotional development, and quality of life.^5^^,^^6^ Previous studies have reported poorer language performance in children with CHD when compared with typically developing peers, with both receptive and expressive language domains potentially affected.^5^^,^^6^ Fourdain et al. reported language vulnerabilities in children with CHD aged 12 to 24 months, reinforcing the importance of early identification of communication delays.^5^ More recently, Provost et al. showed that preschoolers with CHD had lower scores in verbal comprehension, vocabulary acquisition, general ability, and picture naming than healthy controls.^6^ In addition, using functional near-infrared spectroscopy during a passive story-listening task, the authors found reduced activation in posterior temporal regions in children with CHD, and these reduced brain responses were associated with poorer neurodevelopmental outcomes.^6^ Together, these findings indicate that communication difficulties in CHD may reflect both behavioral and neurofunctional vulnerabilities, supporting the need for systematic speech-language surveillance from early childhood.^5^, ^6^, ^7^

In addition to communication difficulties, feeding disorders and dysphagia are common and clinically significant morbidities among children undergoing pediatric cardiac care. Factors such as prolonged endotracheal intubation, postoperative clinical instability, surgical complexity, altered coordination between breathing and swallowing, and possible laryngeal nerve injury may compromise the safety and efficiency of oral feeding.^8^^,^^9^ Feeding impairments may negatively affect nutritional status and growth, but their impact may extend beyond biological outcomes. Feeding is also an important context for oral sensorimotor experience, caregiver-child interaction, communication, and participation in early developmental routines.^10^ Thus, feeding and communication should be considered interrelated functional domains in the hospital care of children with CHD. Beyond their coexistence, both functions share common neuroanatomical and neurophysiological substrates. Feeding, swallowing, vocalization, and early speech production depend on the coordinated activity of overlapping orofacial structures and neural pathways, including the lips, tongue, palate, pharynx, and larynx, under the control of cranial nerves V (trigeminal), VII (facial), IX (glossopharyngeal), X (vagus), and XII (hypoglossal).^11^^,^^12^ These functions also rely on integrated sensory feedback and central pattern generation mechanisms within the brainstem, as well as on the maturation of cortical and subcortical networks involved in sensorimotor control and communication development.^11^^,^^12^ In early childhood, feeding routines also constitute one of the primary contexts for caregiver-child interaction, providing opportunities for social engagement, communicative exchanges, and sensorimotor learning that support emerging language development.^12^^,^^13^ Consequently, postoperative feeding impairments associated with prolonged intubation, recurrent laryngeal nerve injury, altered laryngeal sensation, or swallowing dysfunction may reflect broader vulnerabilities affecting the developmental foundations of communication. From this perspective, feeding difficulties may represent not only nutritional challenges but also early clinical indicators of increased neurodevelopmental risk, reinforcing the importance of integrated surveillance of feeding and communication outcomes in children with CHD.^8^^,^^9^^,^^11^, ^12^, ^13^

Despite increasing recognition of neurodevelopmental vulnerabilities in CHD, most risk models and follow-up strategies remain largely centered on biological, anatomical, and surgical predictors, which may not fully capture the influence of hospitalization-related experiences and early functional performance on developmental outcomes.^3^^,^^7^^,^^14^ Integrating systematic hospital-based screening of feeding and communication development into pediatric cardiac care may therefore provide valuable information beyond conventional clinical and surgical indicators, allowing healthcare teams to identify children at increased developmental risk earlier and support individualized multidisciplinary follow-up strategies. This approach is particularly relevant during hospitalization, when developmental opportunities may be influenced by illness severity, prolonged hospital stays, invasive procedures, feeding limitations, caregiver-child interaction, and variability in care environments.^3^^,^^7^^,^^13^

Therefore, this study aimed to determine the prevalence of communication developmental risk in hospitalized children with congenital heart disease using a standardized screening protocol and to identify clinical, surgical, and hospitalization-related factors independently associated with increased developmental risk. We hypothesized that communication developmental risk would be more strongly associated with functional and hospitalization-related environmental factors than with traditional markers of clinical and surgical severity.

## Material and methods

### Study design and ethical considerations

This cross-sectional observational study was conducted at the Instituto do Coração do Hospital das Clínicas da Faculdade de Medicina da Universidade de São Paulo (InCor-HCFMUSP), a tertiary and quaternary university hospital specializing in cardiovascular care. The institutional Research Ethics Committee approved the study. Legal guardians were informed of the objectives and procedures, and written informed consent was obtained as determined by the Ethics Committee.

### Participants and setting

A convenience sample was adopted, comprising all eligible children hospitalized and evaluated at InCor-HCFMUSP between September 2024 and April 2025 who had been referred to the institution's Speech-Language Pathology Division for assessment and treatment. Participants were identified through the institutional speech-language pathology service database. InCor-HCFMUSP serves pediatric and adult cardiology and heart surgery patients from across Brazil, with 449 inpatient beds (including 158 in intensive care) distributed across seven units.

The Pediatric Service specializes in the management of children and adolescents with highly complex congenital and acquired heart diseases. The pediatric structure includes approximately 43 pediatric/neonatal ward beds, 21 Pediatric Intensive Care Unit (PICU) beds, and six neonatal intensive care unit beds. The pediatric ward admits stable patients who need continuous inpatient monitoring during cardiac care. The semi-intensive care unit cares for patients who require intermediate monitoring and specialized postoperative care. The PICU treats critically ill patients, especially after cardiac surgery, who need ventilatory and invasive hemodynamic support.

The Institution uses a child- and family-centered care model. Caregivers can be present full-time for patients younger than 18-years in all pediatric units. Visitation periods are predefined, with about two hours in ward settings and one hour in intensive care settings. To support child development during hospitalization, the institution offers structured arts, culture, and education activities. A play and cultural environment is located near the pediatric inpatient unit, with books, games, musical instruments, and multimedia resources. Hospitalized children in the pediatric ward routinely access these resources. Semi-intensive care patients may access them as per clinical status. The institution also offers a hospital-based educational service to maintain school engagement and cognitive stimulation during hospitalization. Educational activities are available for hospitalized children aged 1-year or older, based on their clinical condition and care needs.

### Eligibility criteria

Children were eligible for inclusion if they met the following criteria: 1) Clinical and hemodynamic stability at the time of evaluation; 2) Adequate positioning conditions for bedside speech-language assessment; 3) Follow-up by the speech-language pathology team for swallowing-related demands; and 4) Preserved auditory system integrity. Exclusion criteria were: 1) Clinical instability during assessment; 2) Caregivers who were not native speakers of Brazilian Portuguese; and 3) Diagnosis of genetic syndromes. Children with known genetic syndromes were excluded because these conditions are associated with well-characterized, syndrome-specific neurodevelopmental profiles that could independently confound the relationship between hospitalization-related factors and communication risk, which was the primary focus of this study.

A total of 95 children were initially assessed for eligibility. Eleven patients were excluded because they had not undergone cardiac surgery. Among the remaining 84 children, 19 were excluded due to the presence of a genetic syndrome. The excluded syndromic conditions included Down syndrome (n = 12), 22q11.2 deletion syndrome/DiGeorge syndrome (n = 4), Ohdo syndrome (n = 1), Turner syndrome (n = 1), and X-linked syndromic intellectual developmental disorder type 34 (MRX34) (n = 1).

No participants were excluded because of clinical instability during assessment or because their caregivers were not native speakers of Brazilian Portuguese. The final study sample consisted of 65 children who had undergone cardiac surgery and met all eligibility criteria (Fig. 1).

**Fig. 1:**
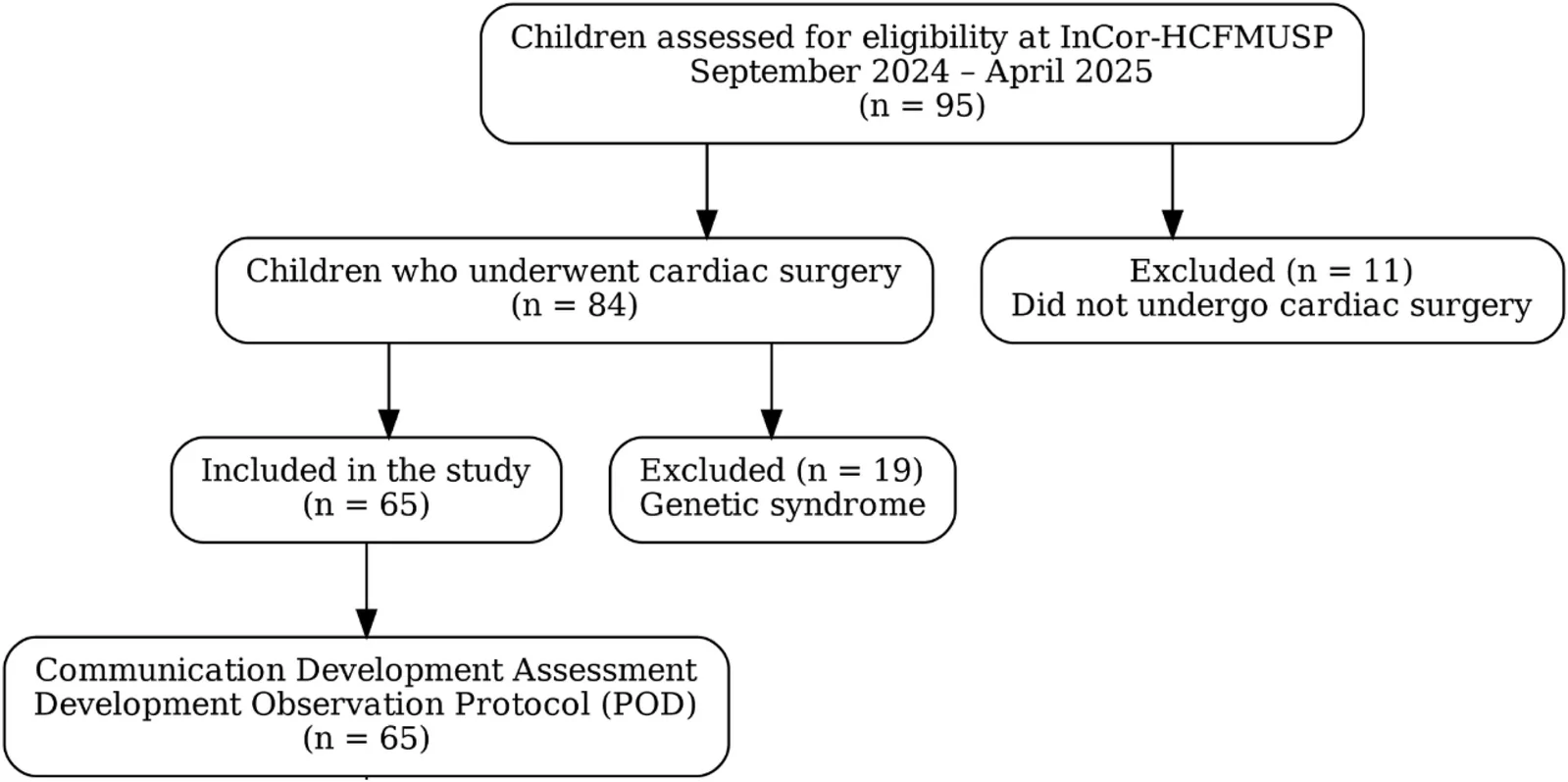
STROBE flow diagram of participant recruitment, eligibility assessment, and inclusion. The flow diagram summarizes the participant selection process. Of the 95 children assessed for eligibility at InCor-HCFMUSP between September 2024 and April 2025, 11 were excluded because they did not undergo cardiac surgery and 19 were excluded due to the presence of a genetic syndrome. The final study sample comprised 65 children who underwent cardiac surgery and completed communication development assessment using the Development Observation Protocol (POD).

### Data collection and procedures

Speech-language evaluations followed the standardized clinical workflow established by the Speech-Language Pathology Division. Participants were identified through active screening of inpatient schedules and the institutional speech-language pathology service database. At the study institution, speech-language pathology assessment is not routinely performed for all children undergoing cardiac surgery; rather, referrals are based on clinical indications, including swallowing-related concerns and recognized risk factors such as prolonged orotracheal intubation or prolonged need for noninvasive ventilatory support. Referral criteria were largely consistent across hospital units, as feeding- and swallowing-related concerns constituted the primary basis for speech-language pathology referral in both intensive care and ward settings, reflecting the current clinical scope of the service.

Eligible children underwent bedside assessment after confirmation of the inclusion criteria. Before the assessment, the electronic medical record was reviewed to obtain relevant clinical information, and the speech-language pathologist contacted the medical or nursing staff before approaching the patient and family. Caregivers received an explanation of the assessment objectives and its role in monitoring communication development during hospitalization. Consequently, the study sample represents a subgroup of children requiring speech-language pathology follow-up during hospitalization and may not be representative of the overall population of pediatric cardiac surgery patients.

Participants' communication development was assessed using the Development Observation Protocol (Protocolo de Observação do Desenvolvimento – POD), a structured speech-language developmental screening instrument published in Brazil.^15^ The protocol follows contemporary developmental monitoring models and family-centered approaches, aiming to support long-term follow-up and identify communication developmental risk early.^15^

The protocol is organized by seven age groups from 0 to 5-years and assesses three domains of development: auditory-linguistic reception, language expression, and oral motor/feeding skills. The POD administration strictly followed the institutional Standard Operating Procedure established at InCor-HCFMUSP, ensuring consistency in data collection procedures across assessments. Scores were summed according to age-specific criteria, then converted to percentages and quartiles.^15^ Communicative performance was classified into four categories: Q1 (high risk), Q2 (risk), Q3 (typical developmental performance), and Q4 (above-average developmental performance). According to the institutional clinical protocol, children classified at or below Q2 were considered at risk for delayed communication development.

POD administration used direct observation of child-caregiver interaction. When needed, play-based interaction with the speech-language pathologist helped supplement behavior observation. Information not directly observed was obtained from caregiver reports, focusing only on the child's current status during hospitalization.^15^

Clinical, surgical, developmental, and feeding-related data were retrospectively extracted from the electronic medical records, including pFOIS scores documented by the speech-language pathology team during routine clinical assessment.

### Variables and clinical classifications

Perinatal variables included gestational age, birth weight, Apgar scores at 1 and 5 minutes, and prenatal diagnosis. Surgical variables included cardiopulmonary bypass duration and aortic cross-clamp time. Postoperative variables included the length of hospital stay. Demographic variables included sex and hospital care units. To ensure data quality and minimize transcription errors, clinical workflows were standardized, and data entry was systematically cross-checked before analysis. Consistency checks were performed throughout data collection and database preparation.

Functional feeding status was assessed using the Pediatric Functional Oral Intake Scale (pFOIS), a validated measure of functional oral intake in pediatric populations.^13^ The pFOIS is an ordinal scale that classifies oral intake according to the degree of oral feeding, feeding restrictions, and dependence on enteral nutritional support, with higher scores indicating greater oral feeding autonomy.^13^ For this study, pFOIS scores were retrospectively extracted from the medical records and corresponded to the feeding status documented by the speech-language pathology team at the time of developmental assessment. The pFOIS score was included as an indicator of functional feeding performance during hospitalization.

Cyanotic heart disease was defined as cardiac defects resulting in arterial oxygen desaturation due to right-to-left shunting or mixing of oxygenated and deoxygenated blood entering the systemic circulation. The anatomical classification of congenital heart disease followed the classification system originally proposed by Clancy et al., as described by Chen et al.,^16^ and was categorized as: I ‒ Biventricular heart without aortic arch obstruction; II ‒ Biventricular heart with aortic arch obstruction; III ‒ Univentricular heart without aortic arch obstruction; and IV ‒ Univentricular heart with aortic arch obstruction.

The surgical risk category was classified according to the Risk Adjustment for Congenital Heart Surgery (RACHS-1) method proposed by Jenkins et al., based on the surgical procedure performed.^17^ The RACHS-1 system categorizes congenital cardiac surgical procedures into six levels according to expected in-hospital mortality risk and surgical complexity, with higher categories indicating greater surgical risk.^17^ For analytical purposes, RACHS-1 categories were grouped into three strata (categories 1–2, category 3, and categories 4–6). This grouping strategy was adopted to preserve the clinical gradient of surgical complexity while reducing sparse-data bias and improving statistical robustness, particularly due to the limited number of patients in extreme-risk categories.^17^

### Data analysis

Statistical analyses were conducted using R statistical software (version 4.6.0 GUI 1.83 High Sierra build 8601; R Foundation for Statistical Computing, Vienna, Austria). The primary outcome was risk for delayed communication development. This outcome was coded as 0 for children without risk (POD > Q2) and 1 for children at risk (POD ≤ Q2).

Initially, exploratory data analysis was conducted, including variable classification, assessment of missing data, and characterization of variables as categorical, ordinal, or quantitative. Categorical variables were described using absolute and relative frequencies and presented as n (%). Quantitative variables were assessed for normality using the Shapiro-Wilk test. Due to the predominantly non-normal distribution and clinical characteristics of the sample, continuous variables were described as median and Interquartile Range (IQR), presented as median [P25–P75].

Categorical variable percentages were calculated based on the number of valid observations. Missing data were handled with available-case analysis for descriptive and comparative analyses. Complete-case analysis was used in multivariable logistic regression analyses.

Comparisons between children classified as “without risk” and “at risk” were performed according to variable type. Quantitative variables were compared using the Mann-Whitney *U test*. Categorical variables were compared using Pearson's Chi-Square test when assumptions were met and Fisher's exact test when expected cell frequencies were insufficient for reliable Chi-Square approximation. Results involving categorical variables with multiple levels and sparse expected frequencies were interpreted cautiously.

The following variables were compared between groups: sex, age, hospital care unit, cyanotic heart disease, gestational age, length of hospital stay, aortic cross-clamp time, cardiopulmonary bypass duration, Apgar scores at 1- and 5-minutes, RACHS-1 score,^17^ birth weight, duration of orotracheal intubation, prolonged intubation (> 48 h), Pediatric Functional Oral Intake Scale (pFOIS),^13^ POD auditory-linguistic reception performance,^15^ POD language expression performance,^15^ and anatomical classification of congenital heart disease.^16^

To investigate factors independently associated with risk of delayed communication development, candidate predictors were selected a priori based on their clinical relevance and previously reported associations with developmental outcomes in children with congenital heart disease. Variables directly related to linguistic, communicative, or developmental performance were not considered for inclusion in the primary multivariable model because these measures contributed to the definition of the outcome itself, potentially introducing methodological circularity.

A multivariable binary logistic regression model was fitted, with Risk group classification as the dependent variable. Independent variables included length of hospital stay, hospital care unit, cyanotic heart disease, gestational age, cardiopulmonary bypass duration, and aortic cross-clamp time. Length of hospital stay, cardiopulmonary bypass duration, and aortic cross-clamp time were modeled in 10-unit increments (10-days or 10-minutes, respectively) to facilitate clinical interpretation of odds ratios. The hospital care unit was treated as a categorical variable, using the intensive care unit as the reference category. A complete-case approach was applied for multivariable analysis; participants with missing data for any variable included in the regression model were excluded. Six participants were excluded due to missing gestational age data, resulting in a final regression sample of 59 children.

Children excluded due to missing gestational age data (n = 6) had significantly older median age (11-months, IQR 9.5–18.5) than those included in the regression model (3-months, IQR 1–6; Mann-Whitney p = 0.002), which may reflect reduced routine documentation of gestational age in older infants and children at the time of hospital admission. No significant differences were observed between excluded and included children regarding risk group classification, hospitalization unit, cyanotic physiology, or perioperative variables, although the small number of excluded cases limits the power to detect subtler differences.

Logistic regression results were reported as adjusted Odds Ratios (ORs), 95% Confidence Intervals (95% CIs), and p-values. Multicollinearity was assessed using the Variance Inflation Factor (VIF), with values > 5 considered suggestive of relevant collinearity. Model discriminatory performance was evaluated using the area under the Receiver Operating Characteristic (ROC) curve.

In the final regression model (n = 59), 22 children (37%) were classified as at risk, corresponding to approximately 3.7 events per variable across the six model parameters, well below the conventionally recommended threshold of 10 EPV, which justified the use of Firth’s penalized logistic regression as a bias correction sensitivity analysis.^18^

Statistical significance was set at p < 0.05. Analyses were considered exploratory, and no correction for multiple comparisons was applied due to the clinical-descriptive nature of the study and sample size. Results were presented using publication-ready tables, comparative graphs, and forest plots to illustrate the main findings.

Because only 25 participants were classified in the risk group, multivariable analyses were considered exploratory and intended to generate hypotheses regarding potential clinical factors associated with developmental risk. Results should therefore be interpreted with appropriate caution.

## Results

### Sample characterization

A total of 65 participants were included in the study. The clinical, demographic, and functional characteristics of the sample are summarized in Table 1. Of the 65 participants, 25 children (38%) were classified into Risk, whereas 40 children (62%) were classified into the No Risk group. The median age of the total sample was 3.0-months, with an interquartile range of 1.0- to 8.0-months. Regarding sex, the distribution was similar across categories: 29 female participants (45%) and 36 males (55%).

**Table 1:** Demographic, clinical, surgical, hospitalization, and functional characteristics of the total sample.

Characteristic	Overall (n = 65)
*Demographic characteristics*	
Sex	
Female (1)	29 (45%)
Male (2)	36 (55%)
Age, months	3 (1, 8)
Gestational age, weeks	38 (37, 39)
Birth weight, g	2940 (2580, 3304.25)
Apgar score at 1-min	
6.0	3 (5.2%)
7.0	12 (20.7%)
8.0	34 (58.6%)
9.0	9 (15.5%)
Apgar score at 5-min	
5.0	1 (1.7%)
7.0	1 (1.7%)
8.0	18 (31%)
9.0	34 (58.6%)
10.0	4 (6.9%)
*Clinical characteristics*	
Cyanosis	
No	20 (31%)
Yes	45 (69%)
Clancy classification	
I	31 (47.7%)
II	4 (6.2%)
III	19 (29.2%)
IV	11 (16.9%)
Risk Classification	
No Risk	40 (62%)
Risk	25 (38%)
*Surgical characteristics*	
RACHS category	
1–2	10 (15.4%)
3	23 (35%)
4–6	25 (38%)
Not Classifiable	7 (10.8%)
Cardiopulmonary bypass time, minutes	26 (0, 156)
Aortic cross-clamp time, minutes	0 (0, 91)
Orotracheal intubation duration, days	5 (1, 15)
Prolonged orotracheal intubation	
No	15 (23%)
Yes	50 (77%)
*Hospitalization and functional characteristics*	
Site	
ICU	18 (28%)
SICU	24 (37%)
Ward	23 (35%)
Length of hospital stay, days	46 (22, 90)
pFOIS	
1	15 (23%)
3	7 (10.8%)
5	24 (37%)
6	19 (29.2%)

Categorical variables are presented as n (%). Continuous variables are presented as median and interquartile range. pFOIS level 2 and 4 were not observed in this sample.

Regarding clinical and surgical characteristics, the median length of hospital stay was 46-days, with an interquartile range of 22- to 90-days. Most children presented cyanosis, 45 cases, 69%, and prolonged orotracheal intubation, 50 cases, 77%. For RACHS classification, the most frequent categories were RACHS 4–6 (38%) and RACHS 3 (35%). The most prevalent Clancy classification was class I (48%), followed by class III (29%).

### Comparison between children with and without risk

The comparison of clinical, demographic, and functional characteristics between children classified as at risk and not at risk is presented in Table 2. Significant differences were observed for site/location, length of hospital stay, and pFOIS. Children in the Risk group were more frequently admitted to ICU and SICU settings, whereas children in the No Risk group were more commonly followed in the ward (p = 0.035).

**Table 2:** Comparison of clinical, demographic, and functional characteristics between the No Risk and Risk groups.

Characteristic	No risk (n = 40)	Risk (n = 25)	p-value
Sex			0.900
Female (1)	18 (45%)	11 (44%)	
Male (2)	22 (55%)	14 (56%)	
Age, months	3.5 (1, 6)	3 (1, 11)	0.200
Site			0.035
ICU (1)	9 (23%)	9 (36%)	
SICU (2)	12 (30%)	12 (48%)	
Ward (3)	19 (48%)	4 (16%)	
Cyanosis			0.500
No	11 (28%)	9 (36%)	
Yes	29 (73%)	16 (64%)	
Gestational age, weeks	38 (37, 39)	38 (36.25, 39)	0.200
Length of hospital stay, days	36.5 (21, 59)	73 (30, 157)	0.014
Aortic cross-clamp time	0 (0, 88.25)	4 (0, 91)	0.900
Cardiopulmonary bypass time	13 (0, 161)	30 (0, 143)	0.900
Apgar score at 1-min			0.400
6.0	3 (7.9%)	0 (0%)	
7.0	6 (15.8%)	6 (30%)	
8.0	23 (60.5%)	11 (55%)	
9.0	6 (15.8%)	3 (15%)	
Apgar score at 5-min			0.400
10.0	3 (7.9%)	1 (5%)	
5.0	0 (0%)	1 (5%)	
7.0	0 (0%)	1 (5%)	
8.0	12 (31.6%)	6 (30%)	
9.0	23 (60.5%)	11 (55%)	
RACHS category			0.900
1–2	6 (15%)	4 (16%)	
3	14 (35%)	9 (36%)	
4–6	15 (37.5%)	10 (40%)	
Not Classifiable	5 (12.5%)	2 (8%)	
Birth weight, g	3044 (2603.75, 3289)	2815 (2557.5, 3330)	0.600
Orotracheal intubation duration	5 (1, 13)	9 (0, 34)	0.800
Prolonged orotracheal intubation			0.500
No	8 (20%)	7 (28%)	
Yes	32 (80%)	18 (72%)	
pFOIS			0.022
1	5 (12.5%)	10 (40%)	
3	5 (12.5%)	2 (8%)	
5	14 (35%)	10 (40%)	
6	16 (40%)	3 (12%)	
Auditory-linguistic receptive performance	0.66 (0.56, 0.83)	0.44 (0.33, 0.56)	<0.001
Expressive language performance	0.54 (0.5, 0.64)	0.38 (0.33, 0.42)	<0.001
Total developmental performance	0.62 (0.56, 0.66)	0.45 (0.38, 0.49)	<0.001
Clancy classification			0.800
I	19 (48%)	12 (48%)	
II	2 (5%)	2 (8%)	
III	13 (32.5%)	6 (24%)	
IV	6 (15%)	5 (20%)	

Categorical variables are presented as n (%). Continuous variables are presented as median and interquartile range. The Mann-Whitney test was used for continuous variables, and the Chi-Square or Fisher's exact test was used for categorical variables. pFOIS level 2 and 4 were not observed in this sample.

The length of hospital stay was significantly longer among children in the Risk group than among those in the No Risk group (p = 0.014), indicating a greater hospitalization burden among children classified as at risk.

The distribution of pFOIS levels differed significantly between groups (p = 0.022). Children in the Risk group were more frequently classified at lower pFOIS levels, whereas the No Risk group showed a predominance of higher pFOIS levels.

Variables related to communicative and developmental performance also differed significantly between groups (Table 2). Children in the No Risk group showed higher auditory-linguistic receptive performance, expressive language performance, and total developmental scores than those in the Risk group (all p < 0.001). These findings should be interpreted cautiously because developmental and communicative measures contributed to the definition of the outcome itself.

The remaining variables analyzed, including sex, age, cyanosis, gestational age, aortic cross-clamp time, cardiopulmonary bypass time, Apgar scores at 1- and 5-minutes, RACHS classification, birth weight, duration of orotracheal intubation, prolonged intubation, and Clancy classification, did not differ significantly between groups.

### Binary logistic regression

To investigate factors independently associated with classification into the Risk group, an exploratory binary logistic regression was performed with Risk status as the outcome. To avoid methodological circularity, variables directly related to linguistic, communicative, or developmental performance were not included in the multivariable model, as these parameters contributed to the definition of the risk group itself.

The multivariable logistic regression model was based on complete-case analysis and included 59 participants. Six children were excluded because gestational age data were unavailable. After adjustment for hospital care units, cyanotic heart disease, gestational age, cardiopulmonary bypass duration, and aortic cross-clamp time, only Ward (compared with ICU) remained independently associated with Risk group classification. Complete model results are presented in Table 3.

**Table 3:** Binary logistic regression for factors associated with the Risk group.

Variable	Adjusted OR	95% CI	p-value
Length of hospital stay	1.05	0.95–1.15	0.340
Site SICU vs ICU	0.80	0.21–3.06	0.747
**Site Ward vs ICU**	**0.13**	**0.02–0.77**	**0.025**
Cyanosis, yes vs no	0.35	0.08–1.46	0.150
Gestational age	0.84	0.63–1.12	0.240
Cardiopulmonary bypass time	1.07	0.94–1.21	0.333
**Aortic cross-clamp time**	0.93	0.75–1.15	0.487

Risk group membership. Results are presented as adjusted odds ratios, 95% confidence intervals, and p-values.

The remaining variables included in the model were not significantly associated with Risk-group classification. Table 3 presents the numerical estimates, and Fig. 2 illustrates the adjusted odds ratios and corresponding 95% Confidence Intervals.

**Fig. 2:**
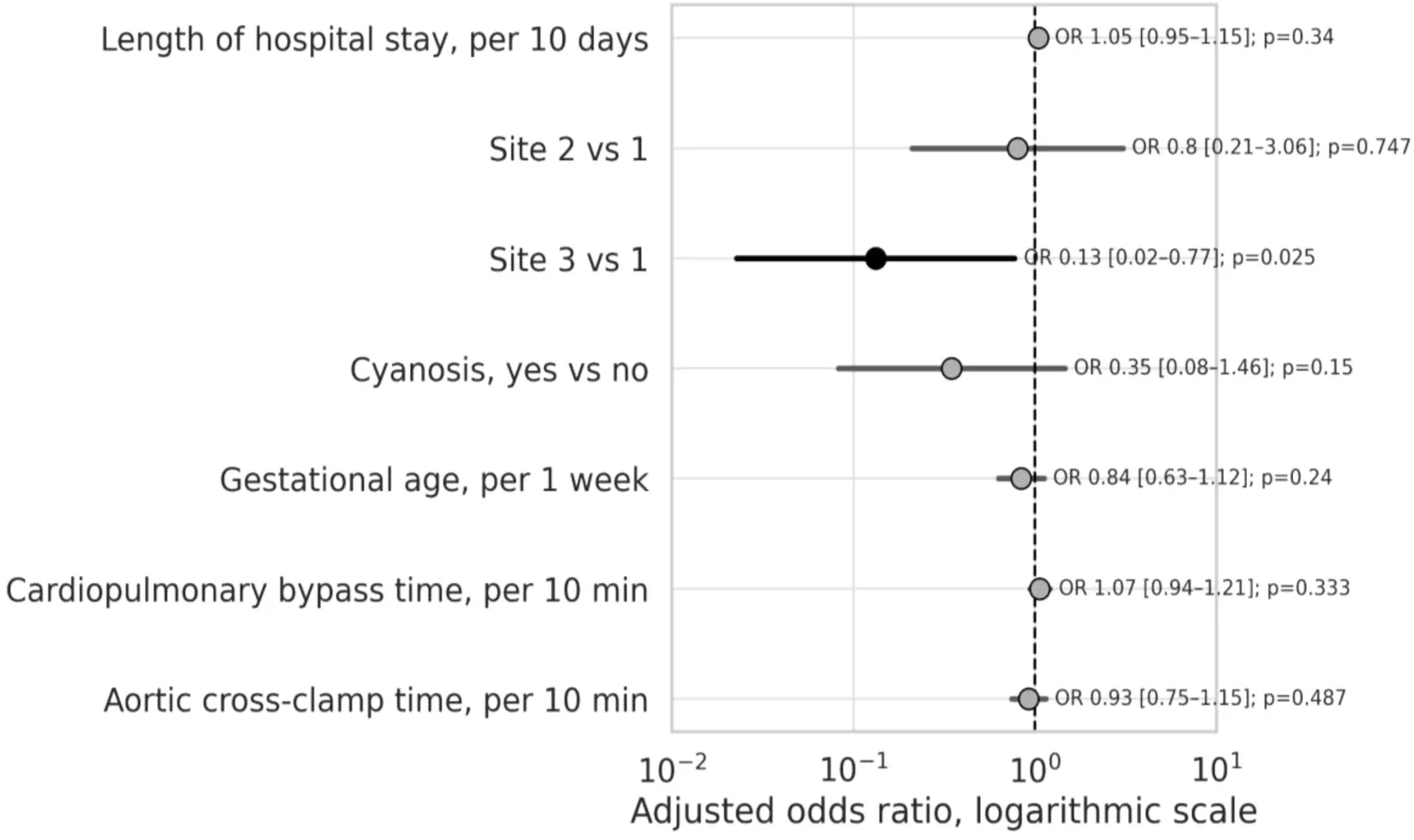
Forest plot of the binary logistic regression model for independent predictors of the risk group. Adjusted odds ratios and 95% Confidence Intervals are shown. The vertical dashed line indicates OR = 1. Values to the left of the line indicate lower odds of belonging to the risk group, whereas values to the right indicate higher odds. Only Ward, compared with ICU, was significantly associated with lower odds of risk group membership.

As a sensitivity analysis, the multivariable model was reanalyzed using Firth's penalized logistic regression to address the low events-per-variable ratio. The results were consistent with those obtained from the conventional model: hospitalization in the ward remained significantly associated with lower odds of risk classification (adjusted OR = 0.18, 95% CI 0.03–0.80, p = 0.024), compared with the conventional model (adjusted OR = 0.13, 95% CI 0.02–0.77, p = 0.025). No other variable reached statistical significance in either model. Complete results of the Firth regression are presented in Table 4.

**Table 4:** Sensitivity analysis: Firth's penalized logistic regression for factors associated with the Risk group.

Variable	Adjusted OR	95% CI	p-value
Site SICU vs ICU	0.84	0.24–2.92	0.782
**Site Ward vs ICU**	**0.18**	**0.03–0.80**	**0.024**
Cyanosis, yes vs no	0.41	0.10–1.50	0.180
Gestational age	0.87	0.66–1.12	0.279
Length of hospital stay	1.04	0.95–1.14	0.372
Cardiopulmonary bypass time	1.05	0.94–1.19	0.368
**Aortic cross-clamp time**	0.94	0.76–1.14	0.534

Risk group membership. Results are presented as adjusted odds ratios, 95% Confidence Intervals, and p-values, estimated using Firth's penalized logistic regression as a sensitivity analysis to address the low events-per-variable ratio in the conventional model.

## Discussion

The main exploratory finding of this study was a statistically significant association between hospitalization in a ward setting and lower odds of communication developmental risk, which persisted after adjustment for the covariates included in the model.

Although biological, clinical, and surgical factors are widely recognized as important determinants of neurodevelopmental risk in children with Congenital Heart Disease (CHD),^1^^,^^2^ length of hospital stay, cyanotic physiology, cardiopulmonary bypass duration, and aortic cross-clamp time were not independently associated with communication developmental risk in the multivariable model. These findings should not be interpreted as evidence that such factors are clinically or biologically irrelevant to neurodevelopmental outcomes in this population. Cyanotic physiology, prolonged cardiopulmonary bypass, and extended aortic cross-clamp time are well-established predictors of brain injury and adverse neurodevelopmental trajectories in children with CHD,^1^^,^^2^ and their absence of statistical significance in the present model most likely reflects the limited statistical power resulting from the small sample size and low events-per-variable ratio, rather than a true absence of association. Under these conditions, the model was underpowered to detect associations of clinically plausible magnitude for variables with modest effect sizes. Future studies with larger, adequately powered samples are needed to determine whether these biological and surgical predictors retain independent associations with communication developmental risk after adjustment for hospitalization-related and environmental variables.

In this context, one possible interpretation is that the hospitalization setting reflects not only differences in clinical severity but also differences in the developmental opportunities available during hospitalization. Within the institutional context of the present study, children admitted to wards have greater access to family presence, recreational and play-based hospital spaces, age-appropriate activities, and opportunities for interaction with peers and healthcare professionals outside the restricted bedside environment. Such conditions may facilitate mobility, play, spontaneous communication, social participation, and engagement in developmentally meaningful experiences. In contrast, the restrictive environment of intensive care units ‒ characterized by prolonged mechanical ventilation, invasive procedures, and limited opportunities for oral motor experience ‒ may deprive children of the progressive sensorimotor experiences that are fundamental to the typical development of both feeding and orofacial communication. The development of swallowing, feeding transitions, and early oromotor precursors to speech relies on cumulative sensorimotor experience from prenatal life through early childhood, and disruptions to this trajectory may affect both the acquisition of feeding skills and the emergence of orofacial communication behaviors.^10^

It is notable that the variable showing the strongest association in the adjusted model was also the one that, within this institutional context, most directly reflects differences in opportunities for interaction and participation during hospitalization. However, given the exploratory nature of the analysis and the low number of events per variable, this observation should be interpreted with caution and not as confirmatory evidence of a causal or protective effect. Therefore, the findings suggest that environmental and relational factors may contribute additionally to the communication risk already associated with the biological vulnerabilities previously described in this population. Opportunities for social interaction, spontaneous communication, family participation, play, and environmental stimulation may be important influences on developmental trajectories in these children. This interpretation is consistent with contemporary developmental care frameworks, which emphasize the importance of relational and environmental experiences in child development.^3^^,^^4^^,^^19^

An alternative, non-mutually exclusive interpretation should also be considered. The hospital care unit at the time of assessment may itself reflect the child's stage of clinical recovery, independent of the severity indicators captured by the variables included in the multivariable model. Children assessed in the ward had typically progressed further in their postoperative recovery trajectory than those still in intensive or semi-intensive care, and this residual clinical recovery status, not fully captured by gestational age, cyanotic physiology, cardiopulmonary bypass duration, or aortic cross-clamp time, could plausibly influence both communicative performance and hospital unit assignment. Therefore, while the environmental interpretation discussed above is consistent with contemporary developmental care frameworks,^3^^,^^4^ the possibility that the hospital unit partly reflects unmeasured clinical recovery status cannot be excluded and should be considered when interpreting this finding.

Crucially, the observed developmental differences between hospital units may also reflect an inherent indication bias deeply embedded in the clinical workflow of tertiary pediatric cardiac care. Although feeding- and swallowing-related concerns constitute the primary basis for speech-language pathology referral across all hospital units, the clinical circumstances prompting these concerns differ systematically by location: children in intensive or semi-intensive care are more often referred for acute, safety-related feeding and swallowing concerns arising during the early postoperative period, whereas children in the ward more often represent a subpopulation that has already progressed through the initial critical phases of recovery, with feeding difficulties reflecting residual rather than acute vulnerability. Consequently, the apparent communicative advantage of the ward group may not solely stem from greater opportunities for environmental stimulation or family interaction; rather, it likely reflects the fact that these children were inherently less neurofunctionally vulnerable at the time of assessment. While this operational constraint limits our ability to completely isolate the independent effect of the physical environment, it does reflect the real-world epidemiological profile of speech-language pathology consultations in a high-complexity hospital setting. This highlights that a child's communication risk profile is dynamic and closely intertwined with their specific stage along the postoperative recovery continuum.

Our multivariable analysis indicated that hospitalization in the inpatient ward was independently associated with a lower likelihood of communication risk (p = 0.025). However, this finding must be interpreted with extreme caution. Due to the small number of events in this specific subgroup, the resulting 95% Confidence Interval was notably wide (95% CI: 0.02‒0.77), which signals model instability due to limited events and a potential overfitting of the regression model. Therefore, rather than a definitive protective effect of the ward environment, this statistical significance may reflect the model instability due to limited events of the model given the limited sample size and low events-per-variable ratio. As a sensitivity analysis, Firth's penalized logistic regression ‒ specifically designed to correct small-sample bias in maximum likelihood estimation ‒ yielded a similar estimate for the ward effect (OR = 0.18, 95% CI 0.03–0.80, p = 0.024) compared with the conventional model (OR = 0.13, 95% CI 0.02–0.77, p = 0.025) (Table 4). This modest attenuation toward the null is consistent with a moderate degree of small-sample estimation bias in the original model, which Firth's correction partially addressed. However, the confidence interval remained similarly wide under both approaches, indicating that the imprecision of this estimate is driven primarily by the limited number of events rather than by estimation bias alone. This sensitivity analysis therefore provides some reassurance regarding the direction and approximate magnitude of the ward effect, but does not resolve the broader limitations related to statistical power, precision, or the indication bias discussed above. Further large-scale studies are required to confirm whether specific environmental factors that differ between the ICU and the ward, such as family presence, opportunities for mobility, natural light exposure, reduced sedation, increased caregiver-child interaction, and lower caregiver stress, translate into true clinical variations in neurodevelopmental risk.^3^^,^^4^

Feeding performance also differed significantly between groups, with children classified as at risk presenting greater feeding restrictions on the pFOIS.^13^ This finding reinforces the hypothesis that feeding and communication represent interrelated functional domains underpinned by shared neuroanatomical substrates (feeding, swallowing, vocalization, and early speech production rely on the coordinated function of overlapping orofacial structures and cranial nerve pathways)^11^^,^^20^ and that disruptions to these systems, as commonly observed in children with CHD following prolonged intubation, laryngeal injury, or surgical complexity,^8^^,^^9^ may reflect broader neurofunctional vulnerabilities extending beyond the oral-motor domain. This interpretation is supported by evidence from broader pediatric populations demonstrating that feeding and dysphagia comorbidities are prevalent among children with developmental delays and persist even among those with isolated language disorders,^21^ and that children with speech and language delay exhibit significantly poorer swallowing performance and oral motor function than typically developing peers even in the absence of neurological conditions.^22^ Particularly relevant from a clinical standpoint is the convergence observed between two methodologically independent instruments: the pFOIS, an internationally validated measure of functional oral intake, and the oral motor/feeding domain of the POD, which contributed to the overall risk classification. The concordance between these distinct tools suggests that restricted functional oral intake during hospitalization may serve as an early clinical indicator of broader neurodevelopmental vulnerability, reinforcing the value of integrating feeding assessment into developmental surveillance protocols.^13^^,^^15^ Although the partial conceptual overlap between instruments warrants caution in interpreting the magnitude of this association as fully independent of the outcome definition, the finding is clinically meaningful and hypothesis-generating. Finally, beyond its neurofunctional significance, feeding also constitutes one of the primary contexts for caregiver-child interaction in early childhood, involving communicative exchanges, sensory experiences, and behavioral regulation,^12^^,^^13^^,^^23^^,^^24^ consequently, feeding restrictions during hospitalization may compound developmental risk by simultaneously limiting both oral motor experience and opportunities for communicative stimulation.

Taken together, the present findings appear most consistent with a multifactorial developmental perspective, in which biological, neurofunctional, and environmental factors act in complementary and interdependent ways. This interpretation is consistent with contemporary models of neurodevelopmental risk in CHD, which recognize the interaction between early brain vulnerabilities, clinical and surgical factors, and environmental experiences throughout development.^1^^,^^2^^,^^14^ It is also supported by neurofunctional evidence in children with CHD: Provost et al.^6^ demonstrated reduced activation of posterior temporal regions during language-processing tasks, associated with poorer neurodevelopmental outcomes, reinforcing the idea that communication vulnerability in this population reflects the interplay of neurobiological, clinical, and environmental factors.

This study has several limitations. The sample consisted of children referred for speech-language pathology follow-up during hospitalization, which may limit the generalizability of the findings to the broader population of children with CHD. Furthermore, as discussed above, although the criterion for speech-language pathology referral was consistent across hospital units, the clinical circumstances prompting referral differed systematically between units, introducing an indication bias that limits the ability to isolate the independent effect of the physical environment on developmental risk. Developmental risk was also identified using a screening instrument rather than comprehensive internationally standardized neurodevelopmental assessments. Additionally, the cross-sectional design precludes both causal inferences regarding the observed associations and assessment of developmental trajectory over time; longitudinal follow-up would therefore be valuable to determine whether communication risk identified during hospitalization persists, resolves, or evolves as children progress through recovery and later development. Finally, the relatively small sample size limits the statistical power of the multivariable analyses.

In particular, as discussed above, the association between ward admission and lower odds of risk classification was based on a small number of events (n = 4 in the ward risk group), and the statistical instability of this estimate underscores the need for replication in larger samples. Furthermore, the events-per-variable ratio in the multivariable model was approximately 3.7, substantially below conventional recommendations, which increases the risk of overfitting and may have contributed to the statistical significance observed for the ward effect independent of its true magnitude. Therefore, the findings should be interpreted as exploratory and hypothesis-generating.

Despite these limitations, the present study provides evidence that communication developmental risk is common among hospitalized children with CHD and suggests that environmental and relational factors may contribute to this vulnerability alongside the biological and clinical factors already recognized. The findings support the incorporation of structured developmental surveillance into routine pediatric cardiac care and reinforce the importance of assessment models that integrate clinical, functional, and contextual dimensions.

From a clinical standpoint, these findings suggest that structured developmental screening need not be reserved for children who have already transitioned to the ward, but could instead be considered earlier in the hospitalization trajectory, including during ICU and semi-intensive care stays. Embedding brief, feeding-focused developmental checkpoints into routine intensive care rounds may allow earlier identification of children who would benefit from targeted, family-centered interventions, such as facilitating caregiver presence, structured play, and graded sensory stimulation as clinical stability permits.^3^

The results also have important implications for intervention planning during hospitalization. If environmental and relational factors contribute to the expression of communication risk, strategies to strengthen caregiver-child interactions may represent promising intervention targets. Recent evidence highlights the important role of caregiver-child interactions in supporting language development and demonstrates that family-centered interventions can improve both children's communication outcomes and caregivers' ability to provide language-enriched environments.^25^, ^26^, ^27^, ^28^

From this perspective, developmental care and family-centered care models become particularly relevant for hospitalized children with CHD. Strategies such as parent coaching, active caregiver participation in daily care routines, shared play, book reading, responsive communication, and structured opportunities for social interaction may increase the availability of developmentally meaningful experiences during hospitalization.^3^^,^^4^^,^^25^, ^26^, ^27^

The findings of the present study do not suggest replacing traditional risk-stratification models based on biological and surgical factors. Rather, they reinforce the need to expand these models by incorporating functional and environmental dimensions that capture additional aspects of neurodevelopmental vulnerability. Future studies should investigate larger, more representative samples, examine the longitudinal predictive value of developmental screening conducted during hospitalization, and determine whether developmental care interventions, family-centered approaches, and environmental modifications can improve communication outcomes in this population.^3^^,^^4^^,^^25^, ^26^, ^27^, ^28^

Despite these methodological limitations, the findings of this study carry meaningful clinical and scientific relevance. By demonstrating that functional and contextual variables were associated with communication developmental risk in a population where established perioperative risk factors did not reach statistical significance, this study contributes to an expanding framework that integrates functional assessment and environmental context into neurodevelopmental surveillance in pediatric cardiac care. The convergence between feeding impairment as measured by the pFOIS and communication developmental risk reinforces that post-surgical dysphagia and feeding vulnerabilities may represent early clinical indicators of broader neurofunctional deficits, rather than isolated nutritional concerns. For multidisciplinary teams at the bedside, this interrelation provides a clinically accessible window for early screening and family-centered intervention before hospital discharge. These findings serve as a hypothesis-generating framework that justifies the integration of structured speech-language screening into routine pediatric cardiology protocols and supports the development of hospital environments that actively foster developmental opportunities during recovery. Confirmation of these findings in larger, prospective studies remains essential before practice recommendations can be established.

## Conclusion

Communication developmental risk was common among hospitalized children with congenital heart disease, affecting more than one-third of the study sample. Hospitalization in a ward setting was independently associated with lower odds of communication developmental risk, whereas traditional markers of clinical severity were not associated with the outcome after multivariable adjustment. Although this finding may reflect differences in developmental opportunities across hospital units, it may also partly reflect differences in clinical recovery status that the model's variables do not fully capture. Feeding performance was also associated with communication developmental risk, consistent with the hypothesis that these functional domains share interdependent developmental mechanisms. Taken together, these results support a multifactorial model of communication vulnerability in children with congenital heart disease and reinforce the importance of integrating structured developmental surveillance into routine pediatric cardiac care. Future studies should investigate larger, more representative samples, examine the longitudinal predictive value of hospital-based developmental screening, and determine whether family-centered developmental care strategies and environmental modifications during hospitalization can improve communication outcomes.

## Declaration of generative AI and AI-assisted technologies in the writing process

During the preparation of this work, the authors used Claude (Anthropic) to assist with revision of the manuscript text and formatting of references according to journal style. After using this tool, the authors reviewed and edited the content as needed and took full responsibility for the content of the publication.

## Authors’ contributions

Camila Pimentel-Piemonte: Responsible for data collection and analysis; interpretation of the results; writing major portion of the paper.

Fernanda Chiarion Sassi: Responsible for supervising the research; interpretation of the results; writing major portion of the paper.

Ana Paula Ritto: Contributed to data analysis and interpretation; organizing the statistical analyses; contributed to manuscript preparation.

Gisele Chagas de Medeiros: Contributed to data collection and data interpretation; contributed to manuscript preparation.

Claudia Regina Furquim de Andrade: Responsible for the research and experimental design; contributed to data analysis and interpretation; contributed to manuscript preparation.

## Funding

This study was financed in part by the Coordenação de Aperfeiçoamento de Pessoal de Nível Superior – Brasil (CAPES) – Finance Code 001.

## Data availability

The data that support the findings of this study are available from the corresponding author upon reasonable request, in accordance with institutional and ethical approval restrictions on patient confidentiality.

## Conflicts of interest

The authors declare that they have no known competing financial interests or personal relationships that could have appeared to influence the work reported in this paper.
